# Differential nasal swab cytology represents a valuable tool for therapy monitoring but not prediction of therapy response in chronic rhinosinusitis with nasal polyps treated with Dupilumab

**DOI:** 10.3389/fimmu.2023.1127576

**Published:** 2023-04-18

**Authors:** Zeynep Danisman, Maximilian Linxweiler, Jan Philipp Kühn, Barbara Linxweiler, Erich-Franz Solomayer, Mathias Wagner, Gudrun Wagenpfeil, Bernhard Schick, Sabrina Berndt

**Affiliations:** ^1^ Department of Otorhinolaryngology, Head and Neck Surgery, Saarlandy University Medical Center, Homburg, Germany; ^2^ Department of Gynecology and Obstetrics, Saarland University Medical Center, Homburg, Germany; ^3^ Department of General and Surgical Pathology, Saarland University Medical Center, Homburg, Germany; ^4^ Department of Medical Biometry, Epidemiology and Medical Informations, Saarland University, Homburg, Germany

**Keywords:** chronic rhinosinusitis with nasal polyps (CRSwNP), biomarker, nasal cytology, precision medicine, type-2- inflammation, Dupilumab

## Abstract

**Introduction:**

Chronic Rhinosinusitis with nasal polyps (CRSwNP) is a common chronic disease with a high impact on patients’ quality of life. If conservative and surgical guideline treatment cannot sufficiently control disease burden, biologicals can be considered as a comparably new treatment option that has revolutionized CRSwNP therapy since the first approval of Dupilumab in 2019. With the aim to select patients who benefit from this new treatment and to find a marker for therapy monitoring, we investigated the cellular composition of nasal mucous membranes and inflammatory cells of patients suffering from CRSwNP and undergoing Dupilumab therapy using non-invasive nasal swab cytology.

**Methods:**

Twenty CRSwNP patients with the indication for Dupilumab therapy have been included in this prospective clinical study. In total, five study visits were conducted with ambulatory nasal differential cytology using nasal swabs starting with the beginning of therapy and followed by visits every 3 months for 12 months. First, these cytology samples were stained with the May-Grunwald-Giemsa method (MGG) and the percentage of ciliated cells, mucinous cells, eosinophil cells, neutrophil cells, and lymphocytes was analyzed. Secondly, an immunocytochemical (ICC) ECP-staining was performed to detect eosinophil granulocytes. Additionally, during each study visit the nasal polyp score, SNOT20 questionnaire, olfactometry, the total IgE concentration in peripheral blood as well as the eosinophil cell count in peripheral blood were recorded. The change of parameters was evaluated over one year and the correlation between clinical effectiveness and nasal differential cytology was analyzed.

**Results:**

In both MGG (p<0.0001) and ICC analysis (p<0.001) a significant decrease of eosinophils was seen under Dupilumab treatment. When patients were divided into a Eo-low- (<21%) and Eo-high- (≥21%) group according to the percentage eosinophils in nasal swab catology in the first study visit, the Eo-high-group showed a greater change of eosinophils over time (Δ17.82) compared to the Eo-low-group (Δ10.67) but, however, no better response to therapy. The polyp score, SNOT20 questionnaire, and total IgE concentration in peripheral blood showed a significant decrease during the observation period (p<0.0001).

**Discussion:**

Nasal swab cytology as an easy-to-apply diagnostic method allows detection and quantification of the different cell populations within the nasal mucosa at a given time. The nasal differential cytology showed a significant decrease of eosinophils during Dupilumab therapy and can therefore be used as non-invasvive method for monitoring therapy success of this cost intensive therapy and potentially can allow an optimized individual therapy planning and management for CRSwNP patients. Since the validity of initial nasal swab eosinophil cell count as a predictive biomarker for therapy response was limited in our study, additional studies including larger number of participants will be necessary to further evaluate the potential benefits for clinical practice of this new diagnostic method.

## 1 Introduction

Chronic rhinosinusitis (CRS) is defined as a persisting inflammation of the sinunasal mucosa which can be further classified into two subtypes depending on whether nasal polyps are present (CRSwNP) or not (CRSsNP) (1). CRSwNP is an increasingly common chronic disease with a prevalence rate of 5-15% in the world-wide population (2–4). Patients suffer from a relevant disease burden with a huge impact on their quality of life and productivity (5). Literature has shown that CRSwNP can affect the patients’ quality of live more severe than congestive heart failure, angina, chronic obstructive pulmonary disease, and back pain, which underlines the high clinical relevance of this disease (6). Symptoms include nasal blockage, nasal discharge, facial pain/pressure, and olfactory dysfunction, usually for more than 12 weeks according to the EPOS 2020 definition of CRS (7). Considering the high prevalence of this disease, the enormous socio-economic costs, and distressing symptoms (8, 9), clinical and basic research aiming to improve surgical and medical treatment of CRS is highly relevant as well as urgently needed due to limited therapy options.

In 80% of all CRSwNP patients in Europe and the US histopathological and molecular diagnostics reveal a type-2 inflammation as major driver of this chronic inflammatory disease (10–12). While the detailed pathogenesis is still not fully understood, recent research suggests a host-environmental hypothesis with a dysfunctional host response, which causes an epithelial barrier dysfunction and leads to a chronic inflammation of the nasal and sinus mucosa (13, 14). The key cytokines in this inflammatory cascade are IL-4, IL-5, and IL-13 inducing a loss of cellular differentiation, reduced junctional integrity, and an impaired innate immune defense (15).

Current guidelines recommend the use of intranasal corticosteroids for at least 4 to 6 weeks as first-line treatment of CRSwNP. If the symptoms maintain under topical steroids, a short course of oral corticosteroids and/or oral antibiotics can be considered (16, 17). If this conservative treatment approach cannot improve the patients’ symptoms and control the disease, functional endoscopic sinus surgery (FESS) is recommended. While a majority of patients show a good response to surgery with a quick relief of clinical symptoms, it is known that the recurrence rate is still high at up to 60%, which frequently necessitates repeated surgeries (18, 19). If both steroid treatment and FESS cannot sufficiently control disease burden and clinical symptoms, biologicals can be considered as a comparably new treatment option that has revolutionized CRSwNP therapy since the first FDA (Food and Drug administration) approval of Dupilumab in 2019. Currently, three biologics are approved for the treatment of CRSwNP: Dupilumab, Mepolizumab, Omalizumab (20). Dupilumab, a monoclonal antibody directed against the IL-4 receptor alpha subunit, inhibits the signaling of the type 2 cytokines IL-4 and IL-13 (21). It demonstrated clinical efficacy and acceptable safety in CRSwNP and other type-2 diseases (e.g. atopic dermatitis and asthma) (22–27) and meanwhile is used in the daily standard of care for patients with severe CRSwNP (28). Additionally, Omalizumab (Anti-IgE) and Mepolizumab (Anti-IL-5) were approved by the FDA as well as the EMA (European Medicines Agency) for medical treatment of CRSwNP patients over the past three years (29, 30).

When considering a biologic as treatement of CRSwNP patients, it is essential to analyze type-2-inflammation biomarkers in order to predict therapy response as well as to monitor treatment success (31). According to current recommendations of EPOS 2020 type-2-inflammation is evident if one can find a blood eosinophil cell count ≥ 250/µl, or a total IgE concentration ≥100 IU/ml, or a tissue eosinophil cell count ≥10/hpf (7). Accordingly, a peripheral blood sample and in many cases also invasive surgical tissue sampling is required for therapeutic decision making. With nasal cytology we herein evaluate a more simple and cost-effective method to study the pattern and profile of different types of cells in the nasal mucosa (32). Nasal cytology has not been tested before in the context of biologic therapy and therefore no comparative literature is available. Against this background, our pilot study aimed to investigate differential nasal swab cytology as a potential tool for monitoring Dupilumab treatment and predicting therapy response to Dupilumab in a monocentric prospective clinical trial including a total of n=20 CRSwNP patients that were monitored with nasal swab cytology over a one-year period (33).

## 2 Material and methods

### 2.1 Patients and tissue samples

**Figure 1:**
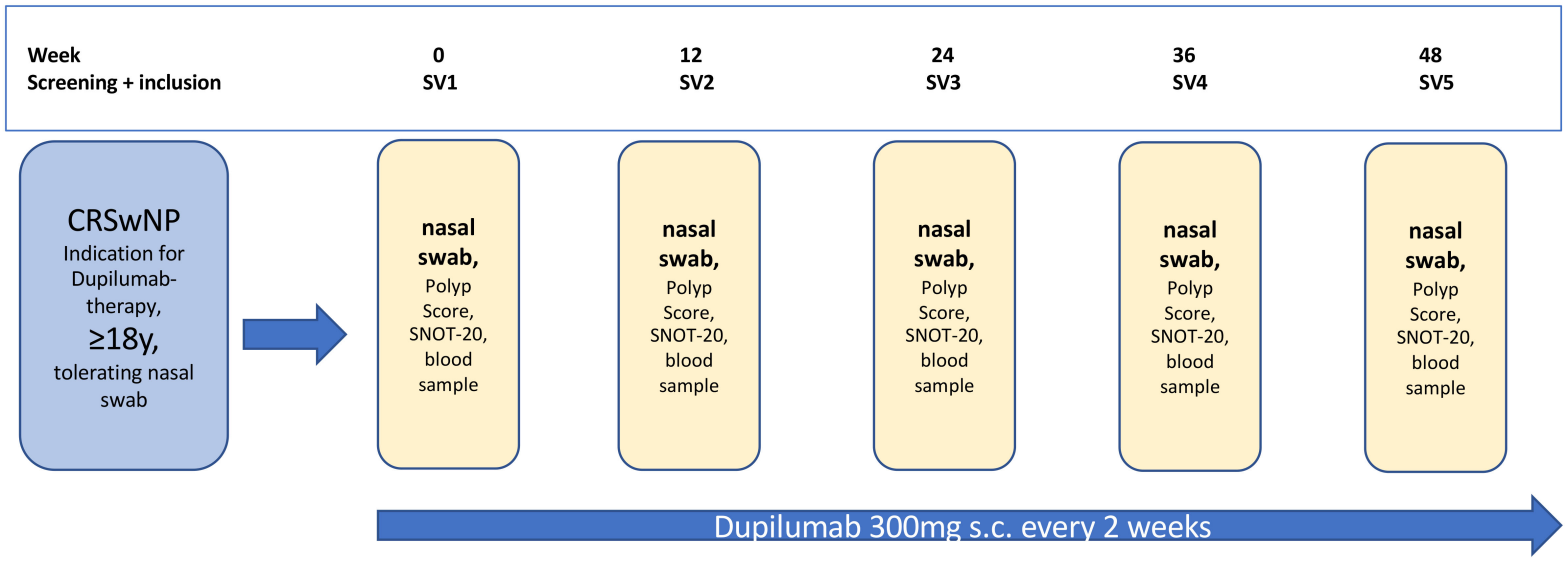
Study flow chart, SV, study visit; SNOT-20, 20-item sino-nasal outcome test; y, years; s.c., subcutaneous.

In our study, we analyzed the change of different parameters over a one-year period in CRSwNP patients who started Dupilumab therapy. The inclusion criteria included medical indication for Dupilumab treatment, age ≥ 18 years, tolerating nasal swab samples, and an informed consent to participate in our study. Referring to this, we totally included n=20 patients between June 2020 and October 2022. All patients were treated at the Department of Otorhinolaryngology, Head and Neck Surgery of the Saarland University Medical Center (Homburg, Germany). Over a period of one year starting with the first Dupilumab application we invited all patients to 5 study visits (SV) as shown in the study flow chart (**Figure 1**). Study visits were schedules at the start of treatement (SV1) and at 12 (SV2), 24 (SV3), 36 (SV4) and 48 (SV5) weeks of treatment. In every study visit, we analyzed the nasal polyp score according to Meltzer et al. (34). The quality of life was measured with the SNOT-20 questionnaire (35), with higher scores indicating a worse quality of life. To detect the serum-IgE and the blood eosinophil cell count we also took a blood sample during every study visit. Olfactometry was performed with the twelve-pen Sniffin’Stick test. If possible, we performed a CT before Dupilumab treatment and evaluated Lund-Mackay CT score. The study was approved by the local ethics review board (Saarland Medicines’ Association Review Board, index number 166/20) and all patients gave their written informed consent to participate in the study. The study was conducted in accordance with the Declaration of Helsinki and all related relevant ethical guidelines.

### 2.2 Nasal swab cytology and MGG-staining

**Figure 2:**
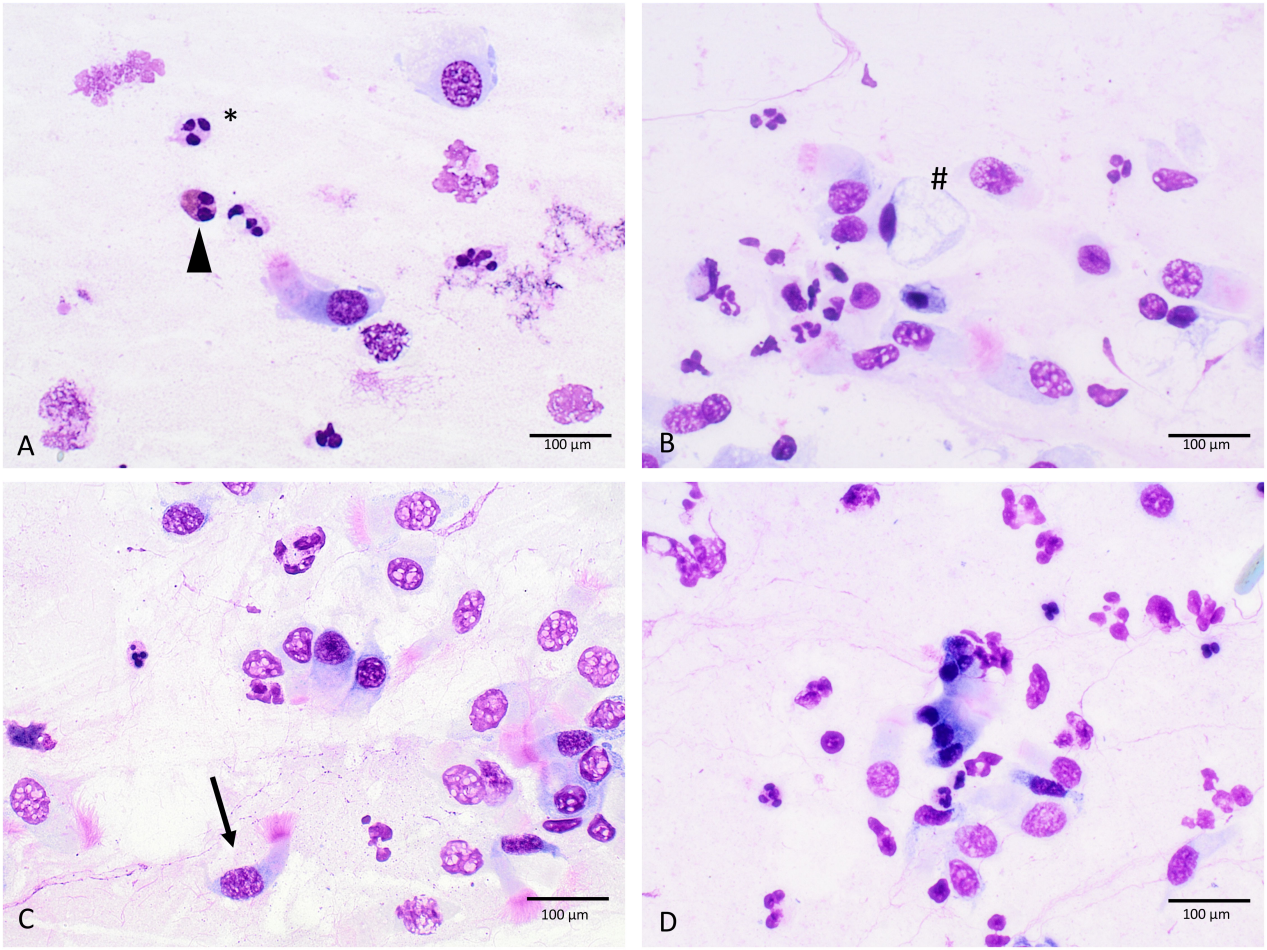
Cellular morphology in MGG-stained slides **(A–D)**, triangle, eosinophilic granulocytes; #, mucinous cell; *, neutrophilic granulocytes; arrow, ciliated cell; 60-fold magnification.

Nasal swab cytology was done within each study visit. Therefore, the mucosa of the inferior turbinate was swabbed with a Medscand cytobrush (Cooper Surgical) by gentle rotating movements as it was expected that the ratio of ciliate/mucinous cells would be well balanced in the middle portion of the inferior turbinate (36). The harvested cells were then directly transferred onto a microscope slide (R. Langenbrinck GmbH, SuperFrost) by wiping off the brush on the glass surface. After drying at room temperature the slides were stained using a modified May Grünwald Giemsa (MGG) method by adding 400 µl acetic acid to the Giemsa solution following a standard protocol (see **Supplementary Table 1**). The stained samples were analyzed using optical microscopy under 60x maginification. In total, 20 high-power fields (hpf) were analyzed for the percentage of ciliated cells, mucinous cells, eosinophils, neutrophils, and lymphocytes (**Figure 2**). The hpfs were selected semi-randomly.

### 2.3 Immunocytology

**Figure 3:**
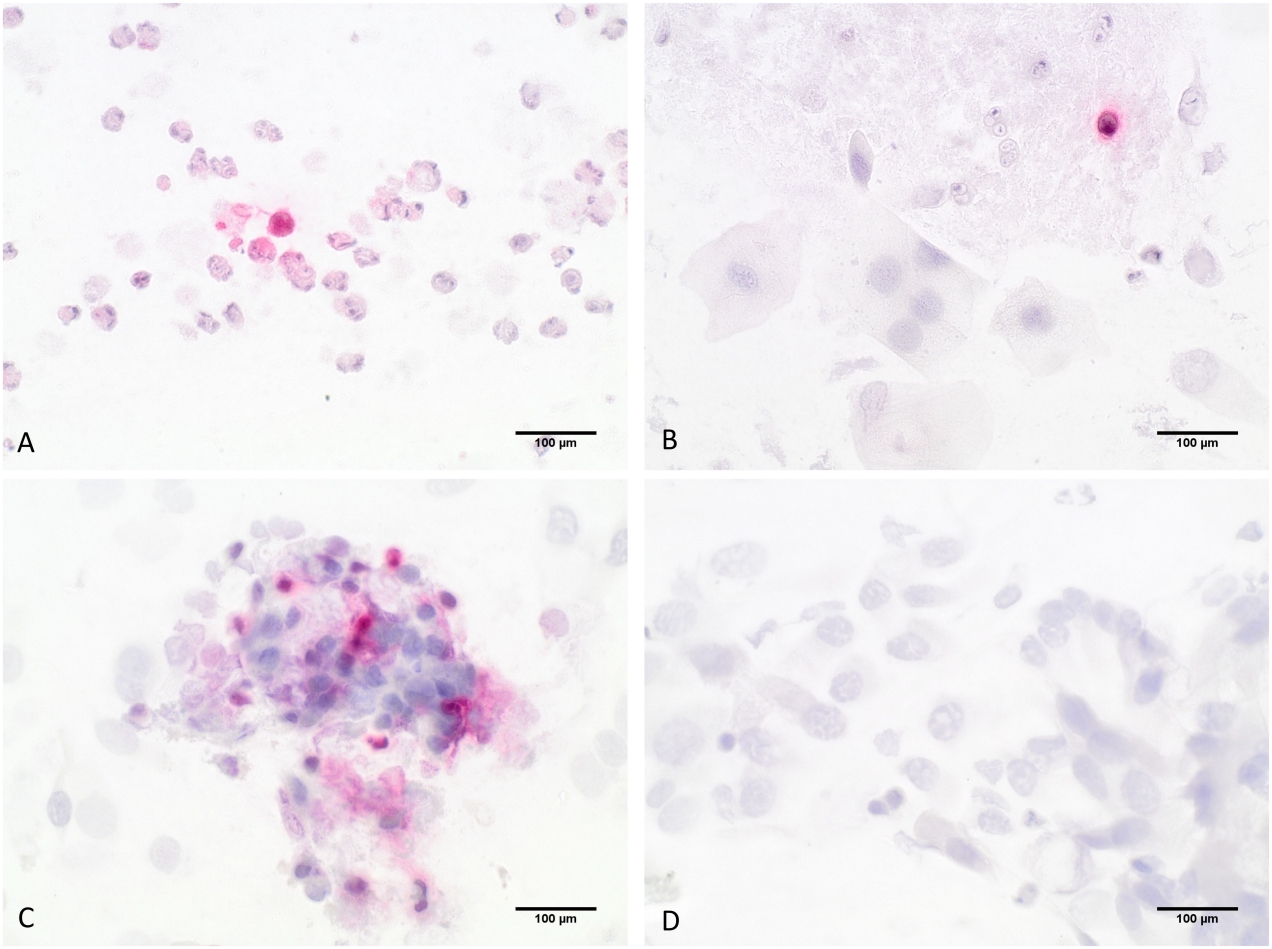
Detection of eosinophils by ICC-staining; **(A–C)** show eosinophilic granulocytes stained in red that were specifically detected by an anti-ribunuclease-3/ECP-antibody, **(D)** shows the negative control slide; 60-fold magnification.

After MGG staining and analysis of the differential cellular pattern, the slides were restained with an immunocytochemical (ICC) ECP- staining to detect eosinophilic granulocytes using an anti-ribunuclease-3/ECP-antibody [EPR20357, abcam]. The staining protocol was first established and optimized using granulocyte concentrates. First, slides were fixated with formalin (4%) for 15 minutes and then heat-induced epitope retrieval was performed by incubating the prepared slides in retrieval buffer (pH 6.0) at 95°C for 20 minutes. Non-specific protein binding sites were blocked with 3% bovine serum albumin (BSA) (Sigma Aldrich) in phosphate-buffered saline (PBS) for 40 minutes at room temperature. In the next step, the slides were incubated for 50 minutes with the primary antibody using a recombinant monoclonal antibody against ribonuclease 3/ECP (1:4000 v/v). Then, visualization was performed using the Dako Real detection system Alkaline Phosphatase (Dako Agilent Technologies, K-5005) according to the manufacturer’s instructions, and the slides were counterstained with hematoxylin (Dako Agilent Technologies). The slides were analyzed using optical microscopy under 60x magnification analyzing 20 hpf and counting the stained eosinophils (**Figure 3**). For each staining series, a granulocyte concentrate slide was used as a positive control, while negative controls were made by omitting the primary antibody in the staining protocol.

### 2.4 Statistics

In the statistical analysis, we tested the change of cell counts over a one-year period in the MGG as well as ICC staining with either a One-way ANOVA or Friedman test using the commercially available software GraphPad Prism 9.4.1 (GraphPad Software, La Jolla, CA, USA) and presuming a significance level of 5% (α = 0.05) as well as a statistical power of 80% (β = 0.8). In testing the significance of thresholds, the existence of normal distribution was controlled by the Kolmogorov–Smirnov test and the Shapiro-Wilk test. If parameters showed no normal distribution, a Friedman test was used. In case of normal distribution, a One-way ANOVA test was used. In the figures, statistical significance levels are indicated with stars (n.s.—non significant; * - *p* < 0.05; ** - *p* < 0.005; *** - *p* < 0.001). All *p*-values < 0.05 were considered statistically significant. To test the difference between two groups (good vs. low responder) a Mann-Whitney U test was used.

## 3 Results

### 3.1 Change of clinical parameters under Dupilumab treatment

Within the 20 patients that were included in our study (14 males, 6 females) the average age was 56 ( ± 13) years. Patient baseline demographics and clinical characteristics are shown in **Table 1**.

**Table 1:** Patient baseline demographics and clinical characteristics; SNOT-20, 20-item Sino-Nasal Outcome Test; NSAID, non-steroidal anti-inflammatory drug; *Higher scores indicate greater disease severity.

(n=20)	Mean (SD) or n (%)
Age	56 ( ± 12)
Sex	
Men	14 (70%)
Women	6 (30%)
Preceding FESS	14 (70%)
Systemic corticosteroid use in the preceding 2 years	14 (70%)
Bilateral endoscopic nasal polyp score* (scale 0-8)	4,5 ( ± 1,85)
SNOT-20 total score* (scale 0-100)	50 ( ± 18)
Lund-Mackay CT score before Dupilumab treatment (n=18)	12,22 (± 4,58)
Baseline blood eosinophils (%)	6,49 ( ± 4,05)
Baseline total IgE (IU/ml)	172,8 ( ± 163,8)
Any type 2 medical history, including asthma or NSAID-exacerbated respiratory disease	19 (95%)
Asthma	18 (90%)
NSAID-exacerbated respiratory disease	4 (20%)
Any type 2 medical history, excluding asthma or NSAID-exacerbated respiratory disease	9 (45%)

**Figure 4:**
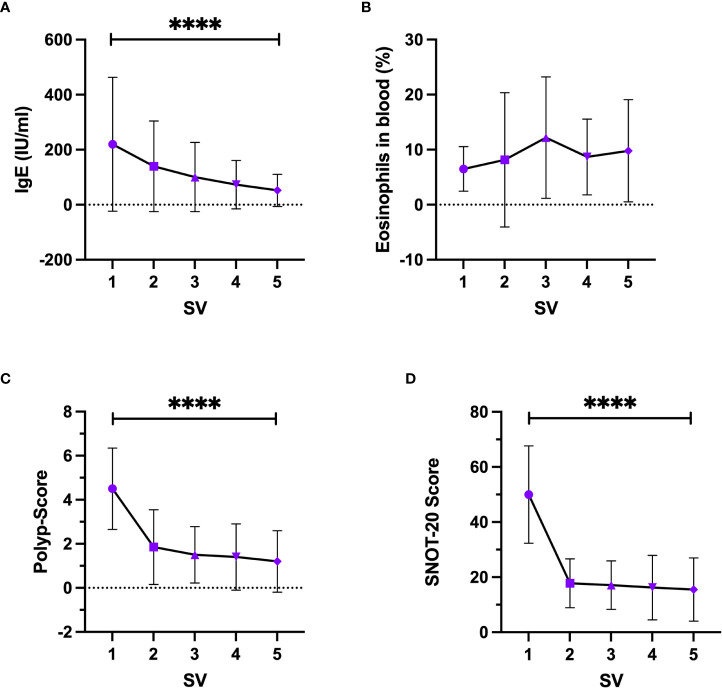
Change of clinical parameters under Dupilumab treatment, **(A)** Serum-IgE, **(B)** Eosinophils in peripheral blood, **(C)** Nasal Polyp-Score, **(D)** SNOT-20 score. SV, study visit; Friedman test, Whisker represents standard deviation, symbol represents mean. ****-p<0.0001.

Serum-IgE levels increased significantly (p<0.0001) over time (**Figure 4A**) with a baseline level at SV1 of 172.8 IU/ml [82.93;262.7] (mean + 95% CI) and a final level at SV5 of 51.37 IU/ml [18.83;83.91]. Eosinophils count in peripheral blood showed no significant change over time (p=0.0786) and varied between 2.9% and 6.9% (**Figure 4B**). The nasal polyp score decreased significantly (p<0.0001) with the greatest change between the first and the second study visit with a change of Δ2.65 (**Figure 4C**). In the first study visit the mean polyp score was 4.5 [3.63;5.37] and decreased up to a mean polyp score of 1.2 [0.55;1.86] in the fifth study visit. The same phenomenon was seen for the SNOT-20 scores (p<0.0001) (**Figure 4D**) with a mean at SV1 of 49.95 [41.68;58.22] and a mean of 15.5 [10.14;20.86] at SV5. The Sniffin’Stick test showed a significant improvement in olfactory function over time (p<0.0001) with an increase from 2.9 [1.13;4.66] at SV1 to 9.11 [7.48;10.73] at SV5.

### 3.2 Development of cellular patterns in swab cytology under Dupilumab treatment

**Figure 5:**
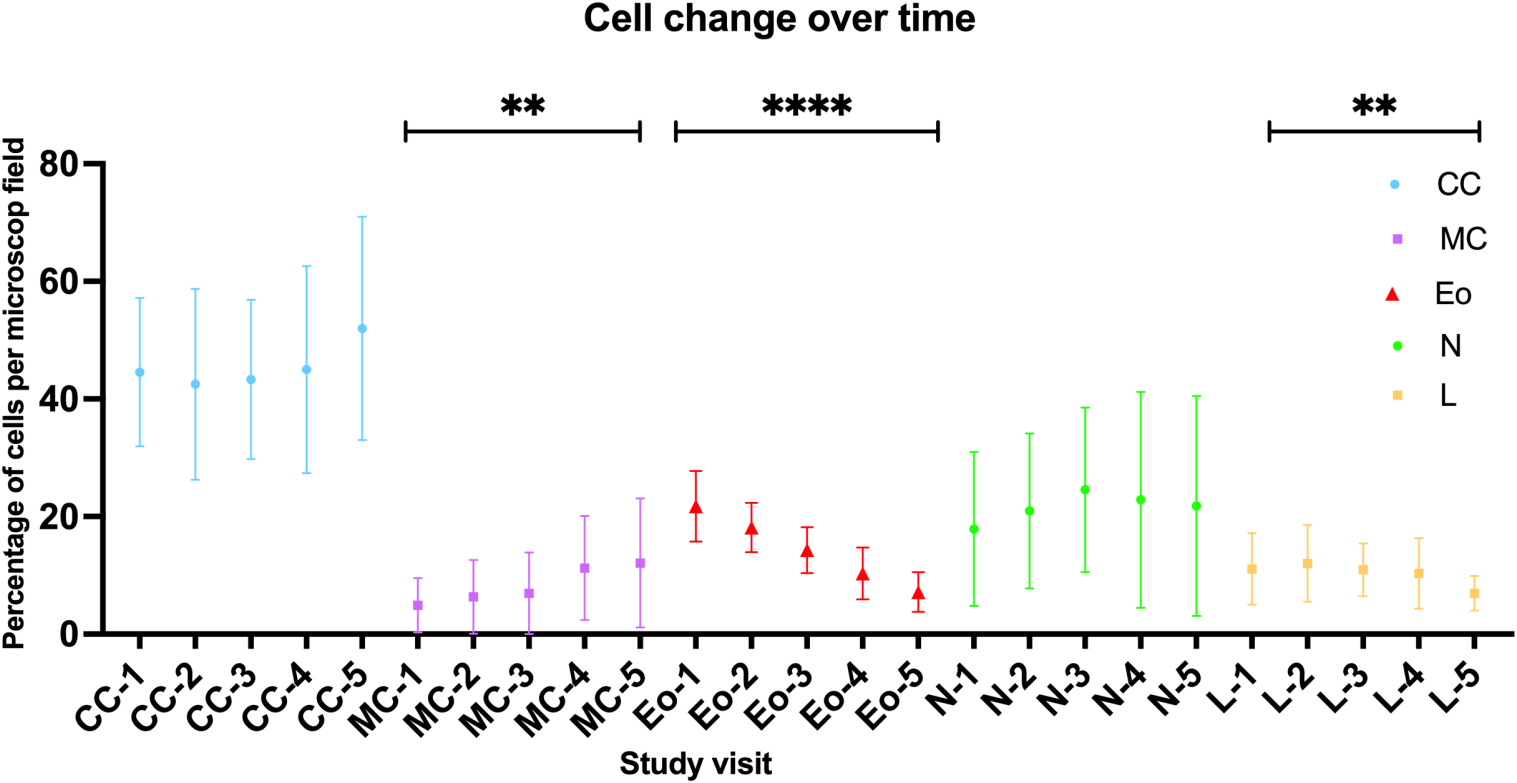
Change of cellular patterns in nasal swab cytology over time as detected by MGG-staining. CC, ciliated cells; MC, mucinous cells; Eo, eosinoiphil granulocytes; N, neutrophil granylocytes; L, lymphocytes; numbers indicate study visit (1-5). For all cells the mean +/- standard deviation is indicated by a symbol with respective error bars; One-way ANOVA/Friedman test. ****-p<0.0001, **-p<0.01.

Results of the MGG-staining are shown in **Figure 5**. The percentage of eosinophils (p<0.0001) as well as lymphocytes (p=0.0082) within the nasal cellular composition decreased significantly over the treatment course. On the contrary, the percentage of mucinous cells increased (p=0.0084). Regarding the neutrophils and the ciliated cells, no significant change under Dupilumab treatment could be found as they varied in all study visits between 15% and 25% (neutrophils) and between 47% and 53% (ciliated cells), respectively. For further analyses, patients were divided into an Eo-high group (≥21%, n=11) and an Eo-low group (<21%, n=9) according to the median of MGG-based eosinophils count in SV1 (21%). In the Eo-high group we found a stronger decrease of eosinophils over the one year follow-up period (ΔEo=17.82%) compared to the Eo-low group (ΔEo=10.67%).

### 3.3 Change in nasal swab eosinophils cell count detected by ICC

After re-staining the same slides that were analyzed after MGG-staining with an immunocytochemical (ICC) ECP-staining method, we re-evaluated the slides and determined the total number of eosinophils in 20 representative hpf. 80% of all slides could be successfully re-stained while 20% of the slides showed no specific reaction in ICC staining due to cellular damage after the re-staining process. Based on ECP-ICC staining, we again found a significant decrease of eosinophils over time (p= 0.0004) comparable to the results described before for MGG-staining. In the first study visit the mean eosinophils cell count was 137.8 [101.2;174.3] and increased to a mean cell count of 47.33 [29.6;65.07] at SV5.

### 3.4 Correlation of pre-therapeutic nasal swab cellular pattern and clinical parameters with therapeutic effectiveness of Dupilumab

In a next step, we divided the patients into a “good-responder” and “poor-responder” group according to the median of the delta changes in nasal polyp-score and the SNOT20 questionnaire score from SV1 to SV5. For the SNOT questionnaire score, patients with a change of 36 or more points belonged to the group of good-responders. For the nasal polyp-score, patients with a change of 3 or more points belonged to the good-responder group. When comparing the initial percentage of eosinophils in nasal swab at SV1 in MGG- and ICC-staining, eosinophils in peripheral blood and total serum-IgE level between the good-responder group and poor-responder group, no significant differences were seen. The correlation between eosinophil cell count in MGG-staining and different parameters analyzed during the study visits (Polypscore, SNOT Score and Serum IgE) and were testet, but no significant results were seen (**Supplementary Figure 1**). Additionally, we compared the ΔSNOT-20 Score, final SNOT Score, ΔPolypscore, final Polypscore, ΔSerum-IgE, and final Serum-IgE between the Eo-low- and Eo-high-group. Herein, we found no significant differences between both groups (**Supplementary Figure 2**).

## 4 Discussion

With the aim to develop a non-invasive and easy-to-use diagnostic tool to monitor clinical effectiveness and potentially predict therapy response to the cost-intensive CRSwNP treatement with Dupilumab, our study investigated the cellular composition of nasal mucosa in CRSwNP patients under Dupilumab treatement with the method of differential nasal swab cytology. In our study, we found a significant change of cellular composition in nasal mucosa under Dupilumab therapy over time indicating a tissue remodeling induced by suppression of type 2 inflammation. Thereby, the eosinophil cell count decreased significantly over time in MGG-stained as well as ICC-based nasal swab cytological analysis. Additionally, the nasal polyp score and SNOT20 questionnaire score decreased significantly indicating a clinically relevant improvement of symptoms. The differentiation between an Eo-low- and an Eo-high-group also highlighted that the difference of changes are stronger in the Eo-high-group, which suggests that Dupilumab is more effective in patients with a higher eosinophil cell number in nasal cytology before treatment though these differences were not statistically significant. Hence, we found a relevant value of differential nasal swab cytology for monitoring therapy response to dupilumab treatment in CRSwNP patients while its value as predicitive biomarker is clearly limited.

Overall, nasal cytology is a useful, inexpensive and easy-to-apply diagnostic method to better examine the nasal mucosa (36). It is repeatable on the same patient, also at short time intervals and an affordable diagnostic technique that can be applied within all age ranges and allows the detection and the quantification of different cell populations within the nasal mucosa at a given time. Previous studies could show that nasal mucosa of healthy patients is usually constituted by four cytotypes (ciliata, mucipara, striata, and basalis) and that the abundance pattern of these cell types can vary under pathophysiological conditions (37). It is also known that CRSwNP patients have a higher eosinophilic cell count in the blood and in polyp tissue (38).

Dupilumab inhibits the signaling of the driving interleukins of a type-2-inflammation and it reduces local type-2 pro-inflammatory biomarkers in CRSwNP (22). As IL-4 and IL-13 activate macrophages, B cells, and epithelial cells to induce recruitment of eosinophils and Th2 cells, their signaling is blocked through the use of Dupilumab (39). Jonstam et al. proved that multiple type 2 biomarker concentrations decreased in nasal secretions and polyp tissue during Dupilumab therapy. They analyzed among others eotaxin-3, total IgE in blood, eosinophilic cationic protein, and eotaxin-2 in nasal polyp tissue (22). These results confirm the results of our study but relied on a surgical and hence much more invasive method of tissue sampling.

In our study we focused on eosinophil cell counts. The prevalence of tissue eosinophil infiltration shows extreme diversity among patients with CRS from Europe, Asia, and the US (40). While patients in Europe and the US show a type-2-inflammation more frequently, patients in Asia show a more differential spectrum. Over the last years there has been observed an eosinophilic shift in Asian countries (10). Myszkowska et al. (41) found a correlation between inflammatory activity and the presence of eosinophils in nasal mucosa with a higher percentage level of eosinophils in more severe inflammation. It is also known that long-term disease recurrence is associated with eosinophil infiltration and IL-5 expression (42). These studies underline that eosinophils are among the most important biomarkers for disease activity and risk of recurrence in CRSwNP patients and should be addressed in biomarker studies due to their potentially high relevance for therapy guidance and management. However, the exact function of eosinophils in the complex network of nasal inflammatory microenvironment is still not fully understood, which necessitates further clinical and preclinical studies to gain a better understanding of eosinophil function in CRSwNP.

We could also see a significant increase of mucinous cells over time. This change in cellular composition could represent a compensation mechanism to equalize the mucosal dryness which is frequently caused by Dupilumab treatment. Barnett et al. found an association between Dupilumab and a relative ocular mucin deficiency (43). A similar phenomenon could be suggested in the nasal mucosa but necessitates further investigation.

Other researchers support a potential clinical benefit of using nasal cytology as a diagnostic tool in rhinology. One of the first published studies in this field investigated the use of non-invasive sampling of nasal cilia for the measurement of beat frequency and the study of ultrastructure more than 40 years ago (44). In 2017, Gelardi et al. developed a clinical-cytological grading of nasal polyps (mild-moderate-severe) to evaluate the chance of surgery (45). With nasal cytology it is also possible to differenciate between different inflammatory patterns of the sino-nasal mucosa that are typically associated with specific diseases (i.e. allergic and non-allergic rhinitis) (46). This information can be useful in cases when other clinical information is not available or nor sufficient to determine the predominantly involved rhinitis phenotype. Nasal cytology can also be used to distinguish between the non-allergic rhinitis forms: those characterized by eosinophilic (NARES), mast cellular (NARMA), mixed eosinophilic-mast cellular (NARESMA) or neutrophilic (NARNE) inflammation (47). Despite this, the diagnostic value of nasal cytology is still underestimated and this technique is still not commonly used in clinical practice.

As of this publication, there is no consensus on the methodology for nasal swab analysis, which is one reason for the lack of uptake of nasal cytology in clinical practice. Furthermore, cyto-morphological experience is necessary to analyze the slides adequately with an otherwise high inter-observer variability in the obtained results (46). One potential approach to overcome these issues is the integration of deep learning techniques and modern semi-automated scanning systems in order to automatically identify and classify cellular subtypes and distribution patterns (48).

Despite the great advantages of nasal cytology as mentioned above, one must be aware that the results of differential nasal cytology analysis can be influenced by several conditions aside from chronic inflammatory processes that represent a potential bias. For example, in case of an bacterial infection higher neutrophil cell counts can be observed. Comparably, one can also find meta- or dysplastic epithelial cells, bacteria, and/or fungal hyphae/spores under special conditions. Additionally one can not exclude that the use of topical medication, e.g. topical steroids, decongestant nasal spray or saline irrigation can significantly alter the cellular pattern in nasal swabs. Therefore, it is essential to study the physiological changes of nasal cytology under the aforementioned conditions in future studies in order to better assess their relevance as potential biases in nasal cyto-morphological diagnostics.

Weaknesses of the current study include the small sample size, due to the relatively rare indications for Dupilumab. Nonetheless, we found highly significant changes in clinical parameters over the course of study visits as well as the cellular pattern in nasal swab cytology even in this limited study cohort underlining the high potential of this technique for a routine clinical use, e.g. for monitoring CRSwNP treatment with biologics and/or choice of therapy. Future studies should look to replicate the findings with larger sample sizes, but also examine the role of nasal swab cytology as a stand alone investigation for diagnosing Type II inflammation.

In conclusion, differential nasal swab cytology is a useful, non-invasive, inexpensive, and easy-to-apply diagnostic method that allows the detection and quantification of cellular populations within the nasal mucosa at a given time. Our study clearly showed that this diagnostic method is suitable as monitoring tool under Dupilumab treatment due to its correlation with clinical findings and patient-reported outcome. However, we found no evidence for a potential benefit of this technique in terms of non-invasively predicting therapy response to Dupilumab. Further studies will be necessary to further evaluate the clinical potential of differential nasal swab cytology for therapy management and clinical decision making in CRSwNP patients.

## Data availability statement

The raw data supporting the conclusions of this article will be made available by the authors, without undue reservation.

## Ethics statement

The studies involving human participants were reviewed and approved by Ethik-Kommission der Ärztekammer des Saarlandes. The patients/participants provided their written informed consent to participate in this study.

## Author contributions

Conceptualization, methodology: ZD, ML, BL, SB, BS, E-FS. Software: ZD, ML. Formal analysis: ZD, ML, JK, BL. Data curation: ZD, ML, SB, JK, E-FS, MW, GW. Writing—original draft preparation: ZD, ML. Writing—review and editing: ZD, ML, SB, BS, E-FS, MW, BL. Visualization: ZD, ML. Supervision: ZD, ML, SB, BS. Project administration: ML, SB. Funding acquisition: ML, BS. All authors have read and agreed to the published version of the manuscript. All authors contributed to the article and approved the submitted version.
