# Supplementary material for: Differential nasal swab cytology represents a valuable tool for therapy monitoring but not prediction of therapy response in chronic rhinosinusitis with nasal polyps treated with Dupilumab
Source: Front Immunol. 2023 Apr 18;14:1127576. doi: 10.3389/fimmu.2023.1127576 (PMC10173305; doi:10.3389/fimmu.2023.1127576)
Supplement: Supplementary file 1 [file DataSheet_1.pdf]

(n=20)	Mean (SD) or n (%)
Age	56 (±12)
Sex<br>Men<br>Women	 14 (70%)<br>6 (30%)
Preceding FESS	14 (70%)
Systemic corticosteroid use in the preceding 2 years	14 (70%)
Bilateral endoscopic nasal polyp score* (scale 0-8)	4,5 (±1,85)
SNOT-20 total score* (scale 0-100)	50 (±18)
Lund-Mackay CT score before Dupilumab treatment (n=18)	12,22 (± 4,58)
Baseline blood eosinophils (%)	6,49 (±4,05)
Baseline total IgE (IU/ml)	172,8 (±163,8)
Any type 2 medical history, including asthma or NSAID-exacerbated respiratory disease	19 (95%)
Asthma	18 (90%)
NSAID-exacerbated respiratory disease	4 (20%)
Any type 2 medical history, excluding asthma or NSAID-exacerbated respiratory disease	9 (45%)
