# Supplementary material for: Differential nasal swab cytology represents a valuable tool for therapy monitoring but not prediction of therapy response in chronic rhinosinusitis with nasal polyps treated with Dupilumab
Source: Front Immunol. 2023 Apr 18;14:1127576. doi: 10.3389/fimmu.2023.1127576 (PMC10173305; doi:10.3389/fimmu.2023.1127576)
Supplement: Supplementary file 2 [file DataSheet_2.docx]

Supplementary Material

# Supplementary Figures and Tables

## Supplementary Figures

**Supplementary Figure 1.** **Correlation between different parameters analyzed during the study visits.** **A**: Polypscore vs. Eos in MGG-staining; **B**: SNOT-20 Score vs. Eos in MGG-staining. **C**: Serum IgE vs. Eos in MGG-staining. Every dot represents one patient. No significant results were seen (Spearman correlation).

Figure 2 Comparison of different parameters between the Eo-low and Eo-high group of patients. A: ΔSNOT-20 Score; B: Final SNOT-20 Score; C: ΔPolypscore; D: Final Polypscore; E: ΔSerum-IgE; F: Final Serum-IgE. The box extends from the 25^th^ to 75^th^ percentiles. The line in the middle of the box is plotted at the median. The whiskers go down to the smallest value and up to the largest. Statistics: A, B, C, E: t-test; D, F: Mann-Whitney U test. No statistically significant differences were seen in any comparison.
