# Supplementary material for: Differential nasal swab cytology represents a valuable tool for therapy monitoring but not prediction of therapy response in chronic rhinosinusitis with nasal polyps treated with Dupilumab
Source: Front Immunol. 2023 Apr 18;14:1127576. doi: 10.3389/fimmu.2023.1127576 (PMC10173305; doi:10.3389/fimmu.2023.1127576)
Supplement: Supplementary file 3 [file Table_1.docx]

**Supplementary table**

**Modified May-Grunwald-Giemsa staining (MGG):**

All steps are done in room temperature.

1. Incubation of the dried slides in May Grunwald solution for 3 min.
2. Air-drying for several seconds.
3. Incubation of slides in Giemsa solution for 20 min.
4. Transfer into a cuvette and syringing under continuous water-flow for 7 min.
5. Air-drying for 24 hours.
6. Stocking of the slides with Entellan up.

Prepared Giemsa solution (not older than 2 days):

25 ml Giemsa + 225 ml phosphate buffer + 400 µl acetic acid
